# Maternal and perinatal Health Research Collaboration, India (MaatHRI): methodology for establishing a hospital-based research platform in a low and middle income country setting

**DOI:** 10.12688/f1000research.24923.3

**Published:** 2021-01-26

**Authors:** Manisha Nair, Babul Bezbaruah, Amrit Krishna Bora, Krishnaram Bora, Shakuntala Chhabra, Saswati S. Choudhury, Arup Choudhury, Dipika Deka, Gitanjali Deka, Vijay Anand Ismavel, Swapna D. Kakoty, Roshine M. Koshy, Pramod Kumar, Pranabika Mahanta, Robin Medhi, Pranoy Nath, Anjali Rani, Indrani Roy, Usha Sarma, Carolin Solomi V, Ratna Kanta Talukdar, Farzana Zahir, Michael Hill, Nimmi Kansal, Reena Nakra, Colin Baigent, Marian Knight, Jenny J. Kurinczuk

**Affiliations:** 1 National Perinatal Epidemiology Unit, Nuffield Department of Population Health, University of Oxford, Headington, Oxford, Oxfordshire, OX3 7LF, UK; 2 Silchar Medical College and Hospital (SMCH), Ghungoor Road, Masimpur, Assam, 788014, India; 3 Mahendra Mohan Choudhury Hospital, Panbazar, Guwahati, Assam, 781001, India; 4 Nagaon Bhogeswari Phukanani Civil Hospital, Haibargaon, Daccapatty, Nagaon, Assam, 782001, India; 5 Mahatma Gandhi Institute of Medical Sciences, Sevagram, Maharashtra, 442102, India; 6 Gauhati Medical College and Hospital (GMCH), Bhangagarh, Guwahati, Assam, 781032, India; 7 Dhubri Civil Hospital, Jhagrarpar, Dhubri, Assam, 783324, India; 8 Srimanta Sankaradeva University of Health Sciences, Narkashur Hilltop, Bhangagarh, Assam, 781032, India; 9 Tezpur Medical College, NH 15, Tezpur, Assam, 784153, India; 10 Makunda Christian Leprosy and General Hospital, Bazaricherra, Karimganj, Assam, 788727, India; 11 Fakhruddin Ali Ahmed Medical College and Hospital (FAAMCH), Barpeta-Hospital-Jania Rd, Joti Gaon, Assam, 781301, India; 12 Jorhat Medical College and Hospital, Kushal Konwar Path, Barbheta, Jorhat, Assam, 785001, India; 13 Institute of Medical Sciences, Banaras Hindu University, Aurobindo Colony, Banaras Hindu University Campus, Varanasi, Uttar Pradesh, 221005, India; 14 Nazareth Hospital, Arbuthnot Rd, Nongkynrih, Laitumkhrah, Shillong, Meghalaya, 793003, India; 15 Assam Medical College (AMC), Barbari, Dibrugarh, Assam, 786002, India; 16 NDPH Wolfson Laboratories, Nuffield Department of Population Health, University of Oxford, Old Road Campus, Headington, Oxford, Oxfordshire, OX3 7LF, UK; 17 National Reference Laboratory, Dr Lal PathLabs, B7 Rd, Block E, Sector 18, Rohini, New Delhi, Delhi, 110085, India; 18 MRC Population Health Research Unit, Nuffield Department of Population Health, University of Oxford, Old Road Campus, Headington, Oxfordshire, OX3 7LF, UK

**Keywords:** Research platform, research model, epidemiology, low-and-middle income country, India, maternal health, perinatal health

## Abstract

**Background:** Maternal and perinatal Health Research collaboration, India (MaatHRI) is a research platform that aims to improve evidence-based pregnancy care and outcomes for mothers and babies in India, a country with the second highest burden of maternal and perinatal deaths. The objective of this paper is to describe the methods used to establish and standardise the platform and the results of the process.

**Methods:** MaatHRI is a hospital-based collaborative research platform. It is adapted from the UK Obstetric Surveillance System (UKOSS) and built on a pilot model (IndOSS-Assam), which has been extensively standardised using the following methods: (i) establishing a network of hospitals; (ii) setting up a secure system for data collection, storage and transfer; (iii) developing a standardised laboratory infrastructure; and (iv) developing and implementing regulatory systems.

**Results:** MaatHRI was established in September 2018. Fourteen hospitals participate across four states in India – Assam, Meghalaya, Uttar Pradesh and Maharashtra. The research team includes 20 nurses, a project manager, 16 obstetricians, two pathologists, a public health specialist, a general physician and a paediatrician. MaatHRI has advanced standardisation of data and laboratory parameters, real-time monitoring of data and participant safety, and secure transfer of data. Four observational epidemiological studies are presently being undertaken through the platform. MaatHRI has enabled bi-directional capacity building. It is overseen by a steering committee and a data safety and monitoring board, a process that is not normally used, but was found to be highly effective in ensuring data safety and equitable partnerships in the context of low and middle income countries (LMICs).

**Conclusion:** MaatHRI is the first prototype of UKOSS and other similar platforms in a LMIC setting. The model is built on existing methods but applies new standardisation processes to develop a collaborative research platform that can be replicated in other LMICs.

## Introduction

Maternal health is a global priority due to the large number of women becoming pregnant every year, an estimated 211 million ^1^, and because of the growing disparity in maternal deaths across countries ^2, 3^. India has the second highest number of maternal deaths with ~45,000 deaths yearly ^2^. The rate is much higher for some states, such as Assam in the Northeast of India. Assam has nearly half the population of the UK, and 6 women die every day as a result of pregnancy and childbirth complications ^4^ compared with around one per week in the UK ^5^. In addition, each year an estimated 5 million pregnant women in India experience a life-threatening complication. To improve care and outcomes, India needs large and robust studies to investigate the risk factors, management and outcomes of pregnancy complications and to find out why disease severity varies from state to state.

In a pilot project (called IndOSS-Assam) we demonstrated the feasibility of setting up a collaborative platform for maternal and perinatal health research jointly undertaken by Indian clinical collaborators and researchers at the University of Oxford ^6, 7^. This was modelled on the UK Obstetric Surveillance System (UKOSS) ^8^ and showed that a hospital-based platform can be used to conduct large epidemiological studies and routine surveys to investigate pregnancy complications and management, and establish incidence and outcomes. UKOSS through its work over the past decade has contributed significantly to improving the safety and quality of care for pregnant women ^8^. It has inspired several high-income countries to establish obstetric surveillance and research systems, which are being used to conduct national and multi-national studies to generate evidence to improve pregnancy care. However, there is no such system in low-and-middle income countries (LMICs) where more than 94% of all maternal deaths occur.

Our pilot work in India not only justified the importance and urgency, but also demonstrated the need to further adapt and improve the pilot model to create a standardised collaborative platform for both research and research capacity building. This led to the establishment of the Maternal and perinatal Health Research collaboration, India (MaatHRI), a larger standardised collaborative research platform of 14 public and private hospitals across four states in India. The objective of this paper is to describe the methods used to establish and standardise the platform and the results of the process. MaatHRI means mother in Sanskrit.

## Methods

MaatHRI is a hospital-based collaborative research platform established to: (i) regularly collect data on the prevalence of known and emerging life-threatening pregnancy complications; (ii) conduct large epidemiological studies to generate evidence to improve maternal and perinatal health in India; and (iii) develop research capacity and skills in the collaborating hospitals. It was built on the pilot system, but extensively expanded and standardised over a period of 18 months from May 2017 to September 2018. The following methods were used to establish the collaborative platform:

1. Establishing a network of hospitals and clinical collaborators
2. Setting up a high-quality secure system for data collection, storage, and transfer
3. Developing a standardised laboratory infrastructure
4. Implementing regulatory systems

### Establishing a network of hospitals and clinical collaborators

Successful completion of the pilot work in two hospitals in Assam allowed us to expand the network from two to nine government hospitals within Assam: six teaching hospitals and four district hospitals. In each hospital, we identified a lead collaborator who were obstetricians. Through their professional networks, we were able to reach out to other hospitals. A hospital was included in the network based on two criteria: (i) willingness of the hospital to participate in a large research collaboration and (ii) a high burden of maternal and perinatal deaths in the population covered by the hospital. Similar to the process used in the pilot work ^6^, we mapped the hospitals to assess the spread and coverage of the population in each state.

### Setting up a high-quality secure system for data collection, storage and transfer

One of the major reasons for success of the pilot work was having dedicated research staff for data collection and data entry. A pragmatic approach was adopted to develop a high quality secure electronic system to overcome the challenges of human resource constraints, lack of dedicated secure computer servers for data storage in the hospitals, and securely sharing data. The following methods were employed:

i. Research nurses were appointed in each hospital and trained
ii. Electronic online data collection forms were developed for entering data
iii. Data are collated automatically in a cloud-based server located in India
iv. Quality assurance and data security procedures were established and implemented

### Standardised laboratory infrastructure

A laboratory infrastructure was created through a partnership with a private laboratory in India, Dr Lal PathLabs (LPL). LPL has a pan-India presence with a network of sample collection centres, regional laboratories and a national reference laboratory in New Delhi, India. Their existing service delivery model was adapted to the requirements of the MaatHRI platform through extensive consultations between the Indian clinical collaborators, and experts at the University of Oxford and LPL. The following services were agreed and are being currently used to standardise the laboratory infrastructure:

- Service 1: Provide blood collection kits with instructions to all study hospitals
- Service 2: Train MaatHRI research nurses to collect and prepare blood samples
- Service 3: Transport samples at ambient conditions from the hospitals to the laboratory
- Service 4: Standardise blood assays
- Service 5: Produce standardised test reports

We tested the model in a run-in phase before full implementation.

### Regulatory systems

The steering committee constituted for the pilot work (IndOSS-Assam) was expanded to form the MaatHRI steering committee. The committee includes representatives from all the collaborating hospitals, the University of Oxford, Indian policy advocates and experts in statistics and ethics. As MaatHRI is a research platform set up to conduct studies on a long-term basis, an independent ‘Data safety and monitoring board’ (DSMB) was set up, including members from India and the UK who are not associated with the MaatHRI platform. A DSMB charter was drafted outlining the roles and responsibilities of the members and how the board will function to provide independent safety review of participants and data, and guidance for observational studies during the course of the ongoing projects. Since the studies currently undertaken through the platform are observational studies, review of adverse event data and reports of serious adverse events (SAEs) are not currently applicable to MaatHRI DSMB. However, should randomised controlled trials be conducted through MaatHRI in the future we would expect the DSMB to be involved in reviewing this type of information.

Academic collaboration agreements were signed between the University of Oxford and all collaborating institutions in India. These were discussed extensively with each institution’s legal team to agree on the terms and conditions including data security and sharing, confidentiality, and intellectual property (IP) rights. To ensure equitable partnership, IP is jointly owned by the collaborating institutions in proportion to the respective contribution of each institution.

### Ethics approvals

Ethics approval was obtained for setting up the platform and to undertake the observational studies from the institutional review boards (IRB) of each coordinating Indian institution, namely: Srimanta Sankaradeva University of Health Sciences, Guwahati, Assam (No.MC/190/2007/Pt-1/126); Nazareth hospital, Shillong, Meghalaya (Ref No. NH/CMO/IEC/COMMUNICATIONS/18-01); Emmanuel Hospital Association, New Delhi (Ref. Protocol No.167); Mahatma Gandhi Institute of Medical Sciences, Sevagram, Maharashtra (Ref No. MGIMS/IEC/OBGY/118/2017); and the Institute of Medical Sciences, Banaras Hindu University, Varanasi, Uttar Pradesh (No.Dean/2018/EC/290). The platform and the studies have also been approved by the Government of India’s Health Ministry’s Screening Committee, the Indian Council of Medical Research, New Delhi (ID number 2018-0152) and by the Oxford Tropical Research Ethics Committee (OxTREC), University of Oxford, UK (OxTREC Ref: 7-18).

## Results

### MaatHRI network of hospitals

**Figure 1.**
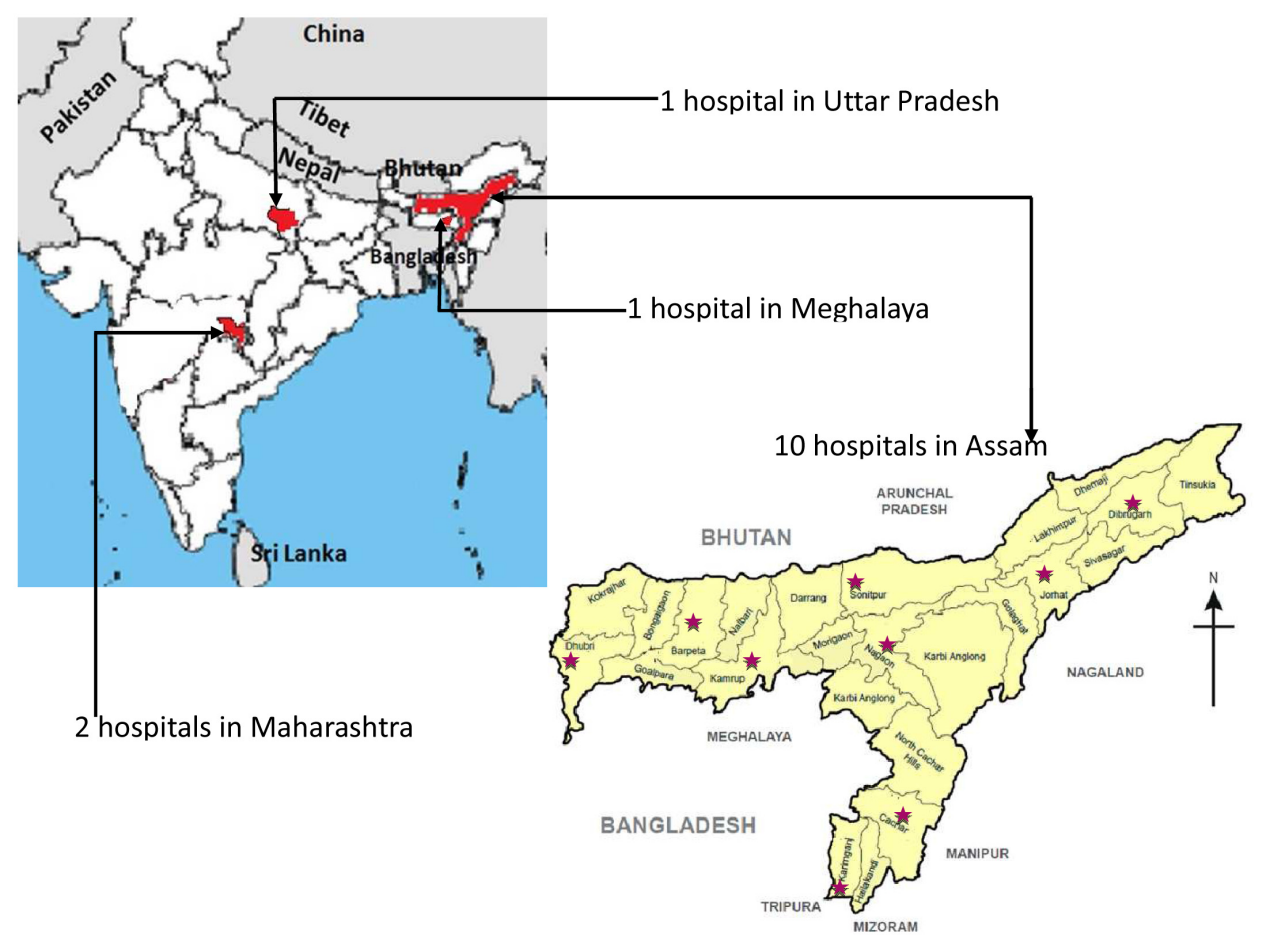
Distribution of the MaatHRI collaborating hospitals and regions covered by the hospitals across India.

We were able to establish a network of 14 hospitals by September 2018 across four states in India – Assam, Meghalaya, Uttar Pradesh, and Maharashtra. After establishing the network, two more hospitals joined MaatHRI, but two government district hospitals left the collaboration. A lack of interest in research and high patient load were the main reasons given by the lead collaborators of the departing hospitals. The MaatHRI platform currently includes a network of 14 hospitals (11 Government and 3 private) who collaborate based on the shared vision of generating scientific evidence to reduce maternal and perinatal mortality and morbidity in India and other LMICs. Figure 1 shows the distribution of the network across India and within the state of Assam. A list of the Indian collaborating institutions are available on the MaatHRI website ( https://www.npeu.ox.ac.uk/maathri). None of the site-collaborators or any clinician involved in the project charge their time contribution to the project. This is another testament of their passion towards the shared vision. The 14 hospitals together conduct about 100,000 deliveries per year. The network includes an Indian research team of 20 nurses, a project manager, 16 obstetricians, two pathologists, a public health specialist, a general physician and a paediatrician.

### Setting up a high-quality and secure system for data collection and storage

*Data and biological sample collection:* Depending on the patient load and related participant recruitment rates, one or two research nurses have been appointed in each collaborating hospital for the MaatHRI work. The nurses are responsible for recruiting study participants, providing participant information and obtaining informed consent, collecting data and blood samples, and following up participants. The research nurses were specifically trained to undertake these activities. In addition, a project manager has been appointed to manage the research nurses and provide supportive supervision.

*Data entry and storage:* Our original plan was for research nurses to collect data in online electronic forms using tablet PCs enabling automatic collation in the Microsoft Azure cloud computing platform (Microsoft Corporation) with servers located in India; there is no provision for storing data on the tablets. However, after an initial trial we found that direct data entry in an online form was not possible due to problems with internet connections in several hospitals and the sensitivity associated with a nurse standing with a tablet PC next to a very sick woman. It was therefore decided that paper forms would be used to collect data in these hospitals and the nurse would enter the data immediately afterwards into the online data portal and then destroy the paper forms. Each hospital has a unique Login ID and password to access the data collection forms and their collated data on the online portal.

*Quality assurance and data security:* The electronic data collection forms have checks and validations to flag logical errors. The project manager is responsible for monitoring data entry on a day-to-day basis. Red flags are raised for errors and incomplete forms immediately so that the research nurse can rectify the errors before the participant is discharged from the hospital. Data stored in the cloud server are encrypted and password protected. Each collaborating hospital can only view and download its own data. Identifiable information are collected for follow-up of participants, but these can only be viewed by the authorised hospital staff and cannot be downloaded by anyone. Once the data collection is complete, in preparation for analysis, all identifiable information is completely delinked from the clinical data to generate pseudonymised analysis files. We have developed secure mechanisms for transferring data within India and between India and the UK with recommended level of end-end-encryption.

### Laboratory infrastructure

Dr Lal Pathlabs (LPL) provides the laboratory infrastructure for MaatHRI. The following services were tested in a trial run before being fully incorporated into the platform.

***Service 1: Blood collection kits with instructions to all study hospitals***. LPL provides the required blood collection kits with specific written guidance to all study hospitals for collecting, processing and packing the blood samples.

***Service 2: Train MaatHRI research nurses to collect and prepare blood samples***. Technical experts from the laboratory trained the MaatHRI staff (project manager and research nurses) to collect, centrifuge and pack samples before the start of studies. When required, a phlebotomist from their collection centre provided supportive supervision to the research nurses during the initial few weeks to correct or prevent any errors.

The MaatHRI research nurses collect, centrifuge and pack blood samples as per instructions in transportation boxes ready for collection by LPL. A standard test requisition form for each participant is filled in by the obstetrician caring for the participant. This form only includes the participant ID, age and a barcode to ensure participant confidentiality and blinding to minimise reporting bias. The test results are only used for research purposes and not for the provision of clinical care.

**Figure 2.**
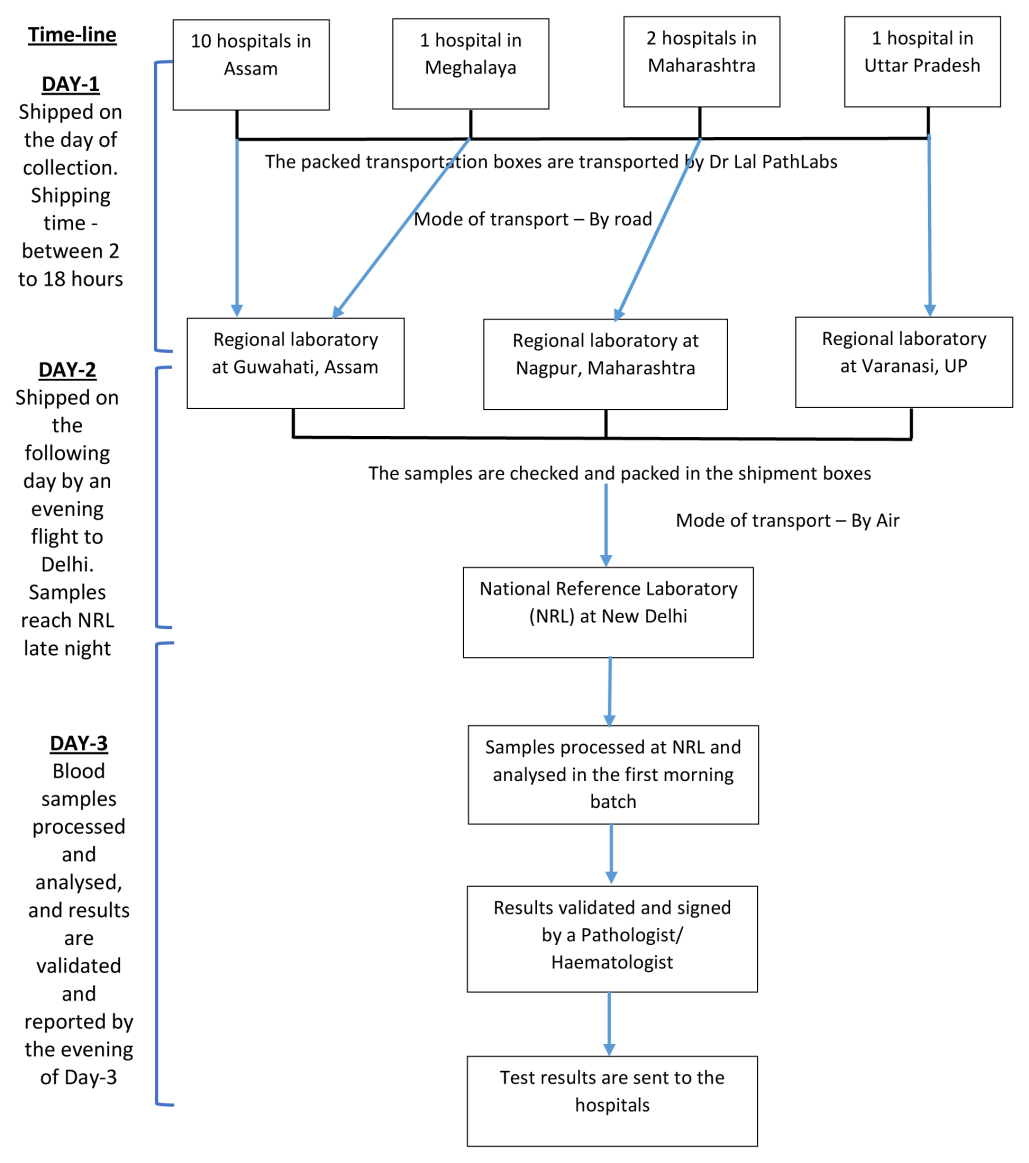
A flow-chart showing the transportation of samples from the hospital to the Dr Lal PathLabs National Reference Laboratory for processing and analysis.

**Figure 3.**
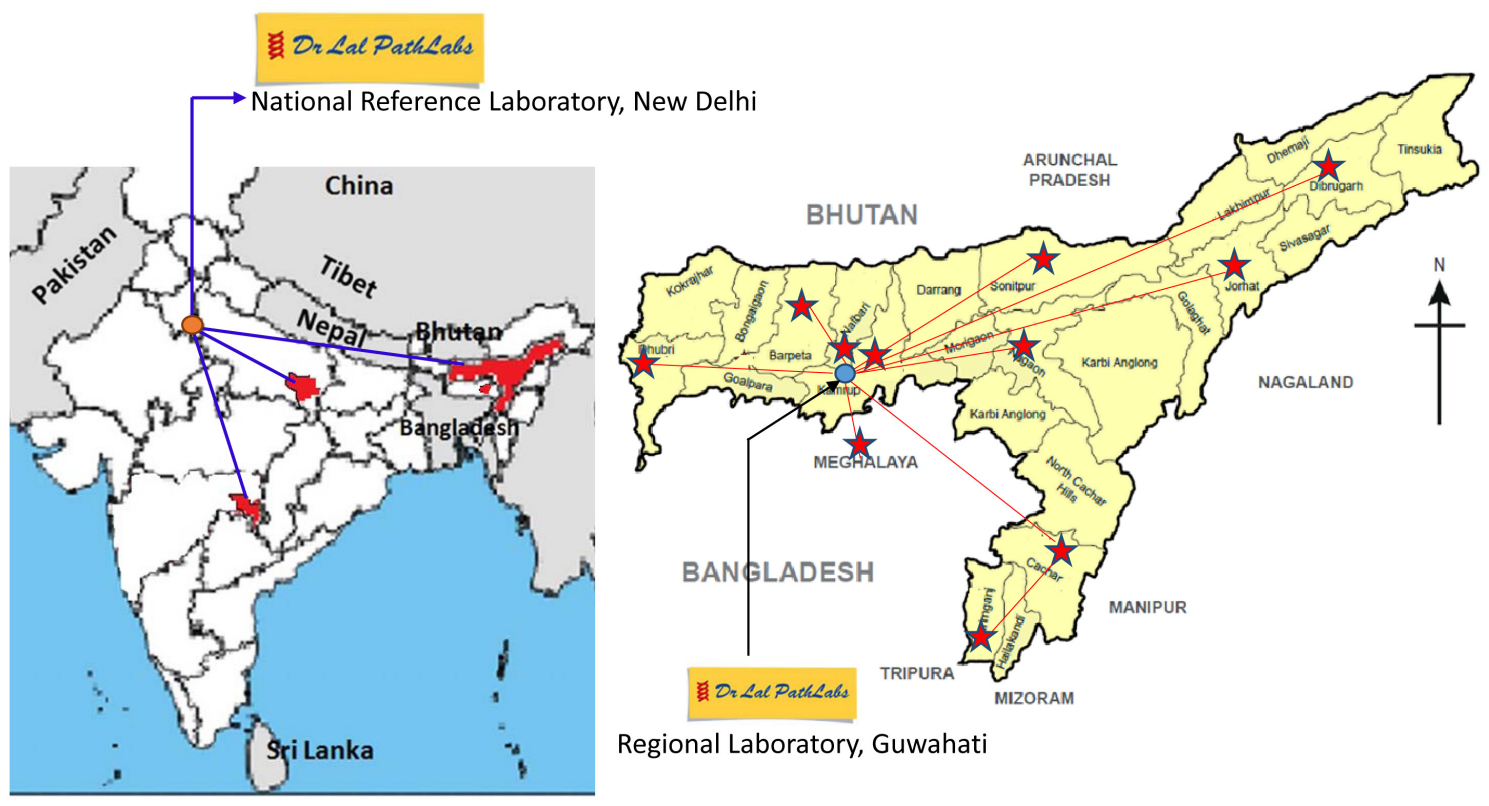
Laboratory network for the MaatHRI platform.

***Service 3: Transport samples at ambient conditions from the hospitals to the laboratory***. A designated person from the LPL collection centre collects the boxes from the hospital. These are transported via road to the nearest regional laboratory where they are checked and then shipped via air to the national laboratory in New Delhi. A flow-chart describing the transportation process from the hospitals to the LPL National Reference Laboratory is shown in Figure 2 and the network is presented in a map in Figure 3. Time in transit is regularly monitored by LPL and reported for each participant along with their test results.

***Service 4: Standardising blood assays***. All samples are processed and analysed in the LPL National Reference Laboratory based at New Delhi. The assay methods, traceability and performance characteristics are discussed by experts from the University of Oxford’s Wolfson laboratory and LPL before including a test in the study. Table 1 shows the traceability and Table 2 shows the performance characteristics for assays that are commonly used for the epidemiological studies undertaken using the MaatHRI platform. The details of specific tests will be presented in subsequent publications. Traceability and assay performance monitoring are important for standardisation of laboratory procedures and quality control. If the quality of a blood sample is compromised in transit, it is not processed, and the site-collaborator and research nurse are advised to collect a fresh sample. The laboratory runs quality control checks daily for each assay (twice a day for some) and monitors their mean coefficient of variation and standard deviation. The results are shared as part of a performance monitoring plan during monitoring and feedback meetings. In addition, LPL also runs a quarterly Quality Improvement Programme.

**Table 1.** Traceability of assays.

Sl No	Name of test	Calibrator traceability (reference material/ reference method)	Units	Typical calibrator value	Calibrator uncertainty of measurement
1	Haemoglobin	1:250 dilution in NCCLS2 recommended reagent for the hemiglobincyanide cyanmethemoglobin	g/dl	12.58	1.00%
2	Hematocrit	Calculated	%	Calculated	NA
3	Platelets	A 1:101 dilution is made using a 20 μL TC pipette and 2 mL of 1% filtered ammonium oxalate (CLSI/ formerly NCCLS)	thou/mm3	214.1	6.00%
4	Serum Ferritin	WHO 3rd International Standard 94/572	ng/ml	Low 5.44 High 953	Low 19.5 High 9.3
5	Haemoglobin electrophoresis	NGSP Certification for A2/F	%	HbF-6.6 % and HbA2-6.7 %	HbF- Low- NA, High 1.8 % HbA2- Low-NA, High- 3.6 %

NGSP - National Glycohemoglobin Standardization Program; CLSI – Clinical and Laboratory Standards Institute; HbF – Fetal haemoglobin; HbA2 - Haemoglobin Subunit Alpha 2; NA - Not applicable

**Table 2.** Assay Information and performance characteristics.

Name of test	System used for the analysis	Method information (supplier/method)	Manufacturers’ analytical range	Laboratory reportable range	Normal reference range (adult woman not pregnant)	Biological variation	Uncertainty of measurement	Quality control material	External quality assurance
Haemoglobin	DxH -800 (Beckman coulter)	Photometric	0.1–25.5	1–25	11.50–15 g/dl	2.5	4.9	Coulter 6c cell control	CAP
Hematocrit	DxH -800 (Beckman coulter)	Automated calculation	Not applicable	Not applicable	36–46%	1.6	3.1	Coulter 6c cell control	CAP
Platelets	DxH -800 (Beckman coulter)	Impedance/coulter principle	3–3000	10–1000	150–450 thou/ mm3	2.6	5.2	Coulter 6c cell control	CAP
Serum Ferritin	Siemens ADVIA Centaur	Chemiluminescence Immunoassay (CLIA)	0.5 – 1650 ng/ml	<0.5, >16500	10–291ng/ml	14.2	22.5	BIO-RAD	CAP PT
Haemoglobin electrophoresis	Variant II Hemoglobin testing system (BIO-RAD)	High Performance Liquid Chromatography	HbF-1.3-44.3 % HbA2-1.6-18.7 %	HbF-1.3-99.8% HbA2-1.6-18.7 %	HbF- <1.5 % HbA2-1.5-3.5 %	HbF-6.8 % HbA2-4.5 %	HbF-13.2 % HbA2-8.8 %	BIO-RAD	CAP

CAP - College of American Pathologists; CAP PT - College of American Pathologists Proficiency Testing programme; HbF – Fetal haemoglobin; HbA2 - Haemoglobin Subunit Alpha

The LPL National Reference Laboratory is accredited by the following bodies – College of American Pathologists (CAP); National Accreditation Board for Testing and Calibration (NABL); British Standards Institution (Quality Management System ISO 9001: 2015, FS 60411).

***Service – 5: Test reports***. Test reports are securely made available to the site-collaborator in each hospital through their usual communication channel. Data from the reports are entered in the electronic forms by the research nurse.

### Regulatory systems

MaatHRI steering committee has met biannually since the platform was established in September 2018. The role of the steering committee is to guide the platform in terms of vision, scope, equitable partnership, and research and training priorities. It is also responsible for communicating the results of the studies undertaken through MaatHRI to the Ministry of Health and Family Welfare (MoHFW), Government of India.

The DSMB periodically reviews participant recruitment, data safety and confidentiality, ethical issues and data quality, ** and examines whether the overall safety and feasibility of the MaatHRI project is acceptable. Although conventionally DSMB is set up for individual studies, we found that setting up a DSMB for the research platform that has oversight of all studies undertaken through the platform could be an effective way to ensure data safety. If the DSMB estimates a potential risk to participant or data security, it will be communicated to the MaatHRI steering committee who has the responsibility to mitigate the problem as soon as possible. The risk and measures taken to mitigate it will also be communicated to all collaborating hospitals and obstetricians as lessons learnt. If the risk or compromise has the potential to cause harm to any participant, the information will be communicated with the participant (at risk) by the site investigator.

The DSMB has met twice since MaatHRI was established in September 2018 and membership includes two obstetricians (one from the UK and one from India), one paediatrician (from India), one biostatistician (from the UK), and one expert in bioethics (from India), all with prior experience and expertise in observational epidemiological studies. They were nominated by the study investigators. So far, no risk or compromise to participant or data security has been identified.

### Studies currently being undertaken through the MaatHRI platform

One survey and three observation studies are currently being undertaken through the platform. A monthly survey of nine life-threatening complications of pregnancy has been in progress since July 2018. The complications are defined using standard definitions and include eclampsia, pre-eclampsia, postpartum haemorrhage, maternal peripartum infection, septic abortion, uterine rupture, heart failure during pregnancy and postpartum, transient peripheral neuropathy, and Japanese encephalitis complications.

The epidemiological studies undertaken are informed by the knowledge and hypothesis generated during the pilot work for IndOSS-Assam. They include: (i) an unmatched case-control study examining the risk factors, clinical characteristics, and outcomes of heart failure in pregnant and postpartum women; (ii) a prospective cohort study investigating the safety of induction and augmentation of labour in pregnant women with anaemia; and (iii) a nested study within the prospective study comparing the coagulation parameters in pregnant women with and without anaemia. Of these, the nested coagulation study is complete, and the other two studies will be completed by June 2022. The monthly survey will continue as long as the collaborative platform exists.

Indian collaborating obstetricians suggest topics for research based on the needs of the local population. For example, based on the observation of high number of cases of health failure during pregnancy, this was proposed as a research topic by one of the collaborating obstetricians. In addition, the conditions included in the monthly survey of pregnancy complications were proposed by the obstetricians. Thus, all research projects undertaken through the platform are co-developed by the Indian and the UK collaborators. Aim of the MaatHRI collaboration is to undertaken research on conditions that adversely affect pregnant women in India so that the results from the studies benefit the future mothers in India and in other LMICs.

## Discussion

MaatHRI, a collaborative research platform, modelled on UKOSS, was successfully established to conduct hospital-based research to improve care and outcomes for mothers and babies in India. It includes 14 public and private hospitals across four states in India, which together conduct about 100,000 deliveries per year. The platform is standardised in terms of data collection, equipment, and laboratory methodology, and employs strict measures for participant confidentiality and data security. It is monitored by two regulatory bodies: a steering committee and an independent DSMB. One survey and three epidemiological studies are being undertaken through the platform.

MaatHRI is the first prototype of UKOSS and other similar platforms ^9^ in a low and middle income country (LMIC). Within this setting, it covers the most deprived and vulnerable population groups. The MaatHRI platform, although built on models of existing surveillance and research platforms in high income countries, is more advanced in terms of using current best practices for standardisation of data and laboratory parameters, monitoring data and participant safety, and secure transfer of data within and between countries. All biological samples are analysed at the LPL National Reference Laboratory. The precision, performance and quality of each laboratory parameter are documented and maintained to a high level. The laboratory partnership also benefits from subsidised costs from LPL for each test, at a rate that is 40% less than their commercial price, with no additional costs for transportation and project management. The laboratory has also started tests for the MaatHRI project, which they did not offer previously. This involved completing extensive validation processes. In addition to high quality and standardisation of the laboratory procedures, the pseudonymised laboratory model ensures confidentiality of participants and minimises reporting bias.

While UKOSS collects anonymised data from hospital records, this is not possible in India and many other LMICs where there are no electronic hospital records system and the paper records are often incomplete. Identifiable information collected locally from participants helps to locate each participant by hospital staff and therefore, another advantage of the MaatHRI platform is the ability to undertake long term follow-up studies of participants. All studies currently undertaken through the platform have a follow-up component with the potential to generate participant cohorts, based on informed consent, for long term follow-up of the effects of pregnancy complications. Adequate measures have been put in place for securely storing the identifiable information and destroying it after the cohorts for long term follow-up have been established. An independent MaatHRI DSMB monitors data safety and participant confidentiality on an ongoing basis, thereby ensuring confidence and trust on the research platform. We are working to achieve a more active and extensive process to involve the public and patients (pregnant women, mothers and their families) and civil societies working to improve the health and wellbeing of mothers and babies in India.

While the platform is established and is currently running three epidemiological studies, the process to develop capacity for research and further improving pregnancy care will continue and is an integral part of the MaatHRI collaboration. The focus is on bi-directional skills development and capacity building through mutual learning between the collaborators in India and the UK. The platform is also being used to develop the research capacity of early career researchers (MSc and PhD students and post-doctoral researchers) interested in working in maternal and perinatal health in an LMIC setting.

### Strengths and challenges

MaatHRI is a collaboration of hospitals that covers deprived populations, some of which are located in remote rural areas of India. While this provides the opportunity to conduct research to improve the health of mothers and babies in areas of the country that have the highest burden of maternal and perinatal deaths, it also poses challenges related to resources and capacity. Appointing new research nurses to collect data and blood samples ensured that the MaatHRI platform was not depriving the hospitals of their scarce human resource. This has created an employment opportunity for nurses in the field of research, which is not a usual job for trained nurses in India. However, the challenge associated with this was the need for extensive training and constant supervision of the nurses. Furthermore, most of the collaborating hospitals had not been involved in a project of this scale and intensity encompassing not just implementation, but designing, standardising and developing the project as equal partners. Therefore, it took more than 20 months of continuous engagement with staff and collaborators to achieve the desired level of quality and standardisation for the MaatHRI platform.

Within the resource constraints, a further challenge is achieving a balance between an ideal collaborative research platform and a pragmatic solution. For example, the ideal platform would have collected data electronically on tablets using online forms, but this was not feasible due to a lack of good internet connectivity in the remote hospitals and cultural sensitivities. Therefore, paper forms are used in some hospitals. However, to mitigate risks and as advised by the DSMB, we have developed a documented process of securely storing and destroying the paper forms within an agreed timeline for each hospital.

Costs related to research staff, standardised laboratory parameters, programming data collection forms, and storing data on Microsoft Azure make studies undertaken through the MaatHRI platform more expensive compared with existing similar systems in the UK ^8^, Europe and Australia ^9^. It is our belief, however, that the benefits of generating high quality scientific evidence to answer important and urgent clinical research questions that will save the lives of thousands of future mothers and babies, outweigh these additional costs.

## Conclusion

In summary, the methods that we have used to develop the MaatHRI platform make it a unique and high-quality research resource using a model that can be replicated in other LMICs. Since being established in September 2018, MaatHRI has already secured further funding, including industry funding. One epidemiological study is complete and two others are in various stages of participant recruitment and data collection. We intend to make the data generated through the MaatHRI platform available to researchers for secondary analysis. We welcome research organisations from India and the UK to use this standardised research platform to undertake studies that are in line with the vision of MaatHRI and will contribute towards reducing the high burden of maternal and perinatal deaths in India. In addition to research impact, our approach to building the platform on the premise of equitable partnership between all collaborators and developing research capacity in the collaborating institutions will further contribute to the sustainability of MaatHRI.

## Data availability

No data is associated with this article.
