# Supplementary material for: Maternal and perinatal Health Research Collaboration, India (MaatHRI): methodology for establishing a hospital-based research platform in a low and middle income country setting
Source: F1000Res. 2021 Jan 26;9:683. Originally published 2020 Jul 7. [Version 3] doi: 10.12688/f1000research.24923.3 (PMC7812614; doi:10.12688/f1000research.24923.3)
Supplement: Peer review history

# Peer review: Reviewer response for version 3

**DOI:** 10.5256/f1000research.54069.r78193

**Published:** 2021-01-26

**Authors:** Anish Keepanasseril

**Affiliations:** 1 JIPMER, Puducherry, India

Thank you for your responses.

Is the rationale for developing the new method (or application) clearly explained?

Yes

Is the description of the method technically sound?

Yes

Are the conclusions about the method and its performance adequately supported by the findings presented in the article?

Yes

If any results are presented, are all the source data underlying the results available to ensure full reproducibility?

No source data required

Are sufficient details provided to allow replication of the method development and its use by others?

Partly

Reviewer Expertise:

Maternal medicine

I confirm that I have read this submission and believe that I have an appropriate level of expertise to confirm that it is of an acceptable scientific standard.

# Peer review: Reviewer response for version 2

**DOI:** 10.5256/f1000research.28694.r76672

**Published:** 2021-01-15

**Authors:** Anish Keepanasseril

**Affiliations:** 1 JIPMER, Puducherry, India

Authors report the method of establishing a collaborative hospital based research platform linking public and private hospitals which could be used as a platform to assess and report various problems of public health importance especially affecting pregnant women.

 As they describe the setting which may have aside variation in the management as well the criteria for the diagnosis of various condition, it needs to describe the strategy to avoid this problem.

 The process of standardisation of the criteria for diagnosis or outcomes needs to be described; was the Delphi consensus or any other approach will be used?

  The UKOSS system is an anonymous voluntary reporting system on the various factors and the outcomes, this methodology paper can also explain the aviation and the difficulties in such as system in a LMIC. 

 It may be worthwhile to consider using this platform to have a biobank project along, as blood sample collected could be stored and later analysed as nest studies if the logistics allow.

Is the rationale for developing the new method (or application) clearly explained?

Yes

Is the description of the method technically sound?

Yes

Are the conclusions about the method and its performance adequately supported by the findings presented in the article?

Yes

If any results are presented, are all the source data underlying the results available to ensure full reproducibility?

No source data required

Are sufficient details provided to allow replication of the method development and its use by others?

Partly

Reviewer Expertise:

Maternal medicine

I confirm that I have read this submission and believe that I have an appropriate level of expertise to confirm that it is of an acceptable scientific standard.

# Response

**Published:** 2021-01-18

**Authors:** Manisha Nair

**Affiliations:** University of Oxford, UK

Authors report the method of establishing a collaborative hospital based research platform linking public and private hospitals which could be used as a platform to assess and report various problems of public health importance especially affecting pregnant women.

Response: We thank the Reviewer for their comments and valuable suggestions.

 As they describe the setting which may have aside variation in the management as well the criteria for the diagnosis of various condition, it needs to describe the strategy to avoid this problem.

Response: We agree with the reviewer that the study hospitals and clinicians could use various criteria for diagnosis and management of pregnancy complication. As mentioned in the results section, “Studies currently being undertaken through the MaatHRI platform”, we use standard international definition and criteria for diagnosing the conditions that are reported in the survey and being investigated in the epidemiological studies. More details about the definitions will be included in the papers that will specifically report the results of the survey and the epidemiological studies.

 The process of standardisation of the criteria for diagnosis or outcomes needs to be described; was the Delphi consensus or any other approach will be used?

Response: We agree with the reviewer and as mentioned above we have standardised the process for diagnosis and investigations including using standard definitions, and standardised laboratory procedures, echocardiography, and data collection tools. We did not use a Delphi process.

 The UKOSS system is an anonymous voluntary reporting system on the various factors and the outcomes, this methodology paper can also explain the aviation and the difficulties in such as system in a LMIC. 

Response: We agree with the reviewer and have added the following in the discussions section of the revised draft:

 “While UKOSS collects anonymised data from hospital records, this is not possible in India and many other LMICs where there are no electronic hospital records system and the paper records are often incomplete. Identifiable information collected locally from participants helps to locate each participant by hospital staff and therefore, another advantage of the MaatHRI platform is the ability to undertake long term follow-up studies of participants.”

 It may be worthwhile to consider using this platform to have a biobank project along, as blood sample collected could be stored and later analysed as nest studies if the logistics allow.

Response: We thank the reviewer for the suggestion. We have been exploring this possibility with laboratories in India. There are no plans at the moment for storing blood samples, but we may be able to do this in future studies using the MaatHRI platform depending on logistics and appropriate regulatory approvals.

# Peer review: Reviewer response for version 2

**DOI:** 10.5256/f1000research.28694.r69549

**Published:** 2020-08-24

**Authors:** Rajmohan Panda

**Affiliations:** 1 Department of Research, Public Health Foundation of India, New Delhi, Delhi, India

Is the rationale for developing the new method (or application) clearly explained?

Partly

Is the description of the method technically sound?

Yes

Are the conclusions about the method and its performance adequately supported by the findings presented in the article?

Yes

If any results are presented, are all the source data underlying the results available to ensure full reproducibility?

No source data required

Are sufficient details provided to allow replication of the method development and its use by others?

Partly

Reviewer Expertise:

NA

I confirm that I have read this submission and believe that I have an appropriate level of expertise to confirm that it is of an acceptable scientific standard.

# Peer review: Reviewer response for version 1

**DOI:** 10.5256/f1000research.27496.r66616

**Published:** 2020-07-27

**Authors:** Rajmohan Panda

**Affiliations:** 1 Department of Research, Public Health Foundation of India, New Delhi, Delhi, India

The authors describe the setting up of a consortium and network for doing observational studies and  RCTs in the future, as such the study does not describe any specific method. It relies more on the aims and objectives of the network and the process followed. The opportunities and challenges are also listed and potential solution in the real world outlined.

 The network is essential for amplifying the work in maternal and new-born health and can serve as a valuable resource for providing evidence and conducting epidemiological studies in these backwards states in India.

  The description could have touched on some of the areas to make it more robust.

 The process of engagement with partners, the MOUs signed and the IPR shared, the shared value systems, the larger universe of health systems and how they plan for risk communication to audiences as well as decision makers including the obstetricians in the network. The incentives and the academic partnerships could have been better described. Often in the north south partnerships, the hospitals and intervention centres are left our to be nothing more than data collectors. Critical questions like how they will benefit, how the population will benefit, do they envisage community panels patient panels and how do they plan to engage them as part of the network. What academic institutes are involved in the global south, what resources do they bring? How does the platform plan for long term sustainability and scalability?

  The ethics part is well described but does not give clarity of was the ethics for setting up the platform or for carrying out the observational studies.

Is the rationale for developing the new method (or application) clearly explained?

Partly

Is the description of the method technically sound?

Yes

Are the conclusions about the method and its performance adequately supported by the findings presented in the article?

Yes

If any results are presented, are all the source data underlying the results available to ensure full reproducibility?

No source data required

Are sufficient details provided to allow replication of the method development and its use by others?

Partly

Reviewer Expertise:

NA

I confirm that I have read this submission and believe that I have an appropriate level of expertise to confirm that it is of an acceptable scientific standard, however I have significant reservations, as outlined above.

# Response

**Published:** 2020-08-07

**Authors:** Manisha Nair

**Affiliations:** University of Oxford, UK

We thank the reviewer for the valuable comments. We have updated the manuscript as advised. Please find below our responses indicating how each comment was addressed. We believe that the revised manuscript has benefited from the comments and suggestions.

Yours sincerely,

Associate Professor Manisha Nair (corresponding author)

Nuffield Department of Population Health, University of Oxford

**Response to the reviewer’s comments**

Comment: The authors describe the setting up of a consortium and network for doing observational studies and  RCTs in the future, as such the study does not describe any specific method. It relies more on the aims and objectives of the network and the process followed. The opportunities and challenges are also listed and potential solution in the real world outlined.

Comment: The network is essential for amplifying the work in maternal and new-born health and can serve as a valuable resource for providing evidence and conducting epidemiological studies in these backwards states in India.

Reply: We thank the reviewer for the comment and for endorsing the value of the MaatHRI platform.

Comment: The description could have touched on some of the areas to make it more robust. The process of engagement with partners, the MOUs signed and the IPR shared, the shared value systems, the larger universe of health systems and how they plan for risk communication to audiences as well as decision makers including the obstetricians in the network.

Reply: We agree with the reviewer that these are important points and have therefore added a the following sections to the ‘methods’ and ‘results’ sections

In methods – under ‘Regulatory systems’ we have added the following paragraph:

“Academic collaboration agreements were signed between the University of Oxford and all collaborating institutions in India. These were discussed extensively with each institution’s legal team to agree on the terms and conditions including data security and sharing, confidentiality, and intellectual property (IP) rights. To ensure equitable partnership, IP is jointly owned by the collaborating institutions in proportion to the respective contribution of each institution.”

We have updated the ‘Regulatory systems’ in the results section as follows:

“If the DSMB estimates a potential risk to participant or data security, it will be communicated to the MaatHRI steering committee who has the responsibility to mitigate the problem as soon as possible. The risk and measures taken to mitigate it will also be communicated to all collaborating hospitals and obstetricians as lessons learnt. If the risk or compromise has the potential to cause harm to any participant, the information will be communicated with the participant (at risk) by the site investigator.  

The DSMB has met twice since MaatHRI was established in September 2018 and membership includes two obstetricians (one from the UK and one from India), one paediatrician (from India), one biostatistician (from the UK), and one expert in bioethics (from India), all with prior experience and expertise in observational epidemiological studies. They were nominated by the study investigators. So far, no risk or compromise to participant or data security has been identified.”

The collaboration is based on the shared vision of generating scientific evidence to reduce maternal and perinatal mortality and morbidity in India and other LMICs. We have now stated this clearly in the ‘Results section’ under ‘MaatHRI network of hospitals’.

Comment: The incentives and the academic partnerships could have been better described. Often in the north south partnerships, the hospitals and intervention centres are left out to be nothing more than data collectors. Critical questions like how they will benefit, how the population will benefit, do they envisage community panels patient panels and how do they plan to engage them as part of the network.

Reply: These are crucial points and I thank the reviewer for raising them. We absolutely agree that there could be power imbalance between collaborating organisations. This is not only true for international collaborations between the North and South, but also between academic collaborations within a country. We have made especial efforts through the regulatory systems to make the partnerships equitable and as already mentioned in the discussion section, our focus is on bi-directional skills development and capacity building through mutual learning between the collaborators in India and the UK.

Indian collaborating obstetricians are the ones who suggest topics for research based on the needs of the local population. For example, based on the observation of high number of cases of health failure during pregnancy, this was proposed as a research topic by one of the collaborating obstetricians. In addition, the conditions included in the monthly survey of pregnancy complications and death were decided by the obstetricians. Thus, all research projects undertaken through the platform are co-developed by the Indian and the UK collaborators. Since the research undertaken is only on conditions that are known to affect pregnant women in India, the results from the studies will benefit the future mothers in India as well as mothers in other LMICs. We have now clearly stated the above in the results sections of the revised article in the section ‘Studies currently being undertaken through the MaatHRI platform’.

We are working to achieve a more active and extensive process to involve the public and patients (pregnant women, mothers and their families) and civil societies working to improve the health and wellbeing of mothers and babies in India. This has been added to the discussion section.

Comment: What academic institutes are involved in the global south, what resources do they bring?

Reply: As advised by the reviewer, we have added the following sentence to the results section: “A list of the Indian collaborating institutions are available on the MaatHRI website ( https://www.npeu.ox.ac.uk/maathri).”

In addition to co-developing the studies, another major resource that the collaborators contribute is their time. None of the site-collaborators or any clinician involved in the project charge their time contribution to the project. This is another testament of their passion towards the shared vision. This information is also added in the revised version.

Comment: How does the platform plan for long term sustainability and scalability?

Reply: This is an important point and some details were already included in the conclusion section. We have further clarified this by adding the following: “We welcome research organisations from India and the UK to use this standardised research platform to undertake studies that are in line with the vision of MaatHRI and will contribute towards reducing the high burden of maternal and perinatal deaths in India.”

Comment: The ethics part is well described but does not give clarity of was the ethics for setting up the platform or for carrying out the observational studies.

Reply: As advised by the reviewer, we have reworded sentences to make this more clear: “Ethics approval was obtained for setting up the platform and to undertake the observational studies from……”

“The platform and the studies have also been approved by the Government of India’s…..”
